# Testing the Material Time Concept in Nanoconfined Glasses: Equilibration Kinetics of 5PPE in Nanopores

**DOI:** 10.1021/acs.jpcb.6c02082

**Published:** 2026-08-18

**Authors:** Zuzanna Pytlik, Gopika Mohandas, Karolina Skrzydelska, Katarzyna Chat, Karolina Adrjanowicz

**Affiliations:** Institute of Physics, Faculty of Science and Technology, University of Silesia in Katowice, 75 Pulku Piechoty 1a, 41-500 Chorzów, Poland

## Abstract

Glassy materials undergo physical aging, changing their physical properties as they approach thermodynamic equilibrium. When confined in nanopores, glass-forming systems exhibit peculiar behavior, including out-of-equilibrium phenomena observed on a molecular time scale much faster than in the bulk. This occurs during rapid cooling, when the fraction of molecules located near the confining walls becomes kinetically frozen at the experimental time scale. As a result, the α-relaxation of the core fraction becomes faster than the equilibrium bulk liquid at the same temperature. Upon prolonged annealing, confinement-induced changes in the glass transition dynamics can be progressively eliminated, and the α-relaxation time recovers its bulk value. Recently, it has been demonstrated that a “material time” concept—the idea that structural recovery is governed by an internal clock whose rate changes as the system ages—can capture the out-of-equilibrium response of nanoscale confined glasses. Here, we test this framework for a model van der Waals liquid, 5-ring polyphenyl ether (5PPE), confined within self-ordered nanoporous alumina templates of straight cylindrical nanochannels. We show that the equilibration kinetics can be successfully predicted for pores of different sizes and various thermal protocols. Our results provide new experimental evidence that the single-parameter aging concept remains valid not only for macroscopic glasses but also for nanoscale-confined glass-forming liquids.

## Graphical abstract

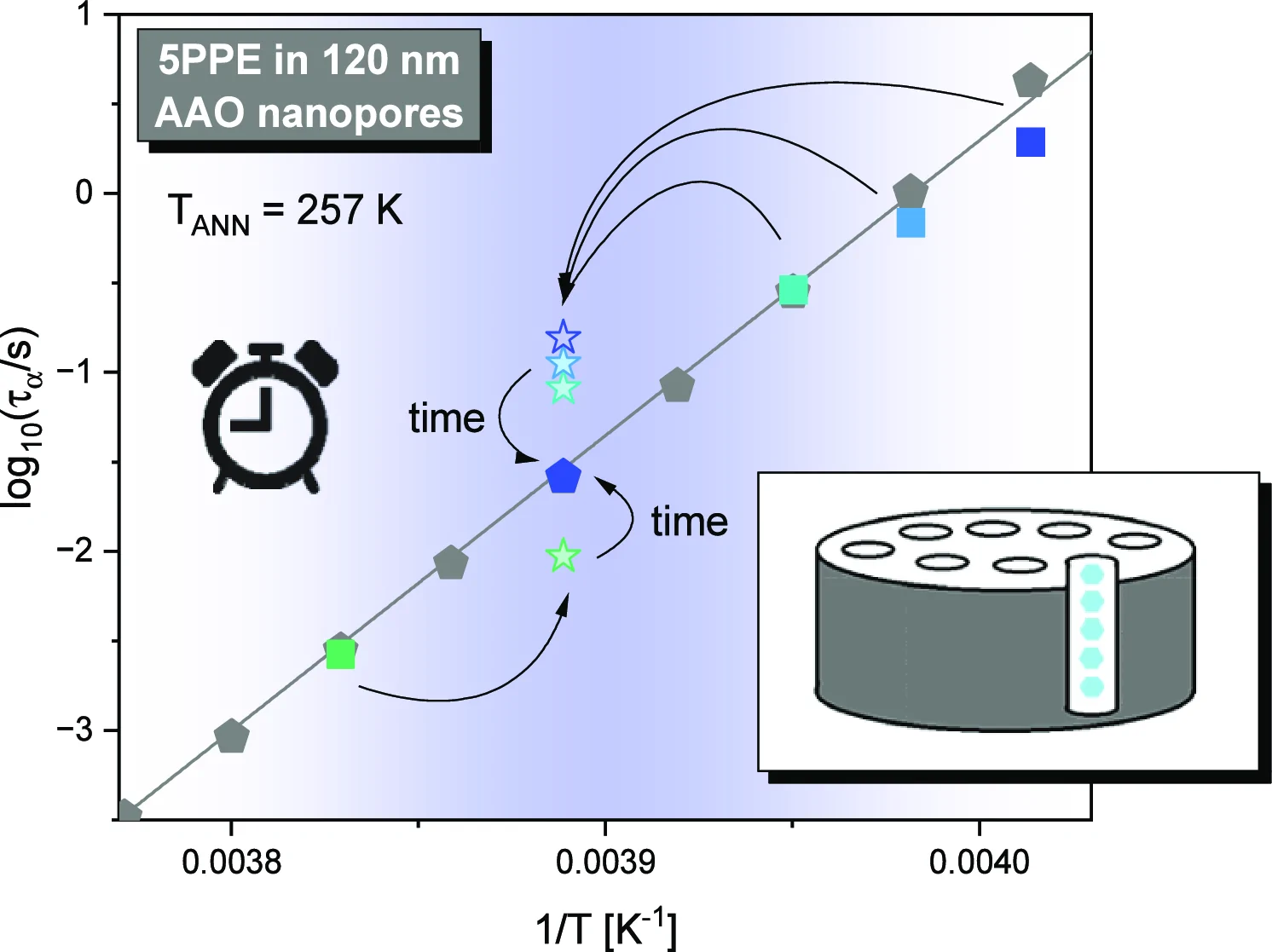

## 1 Introduction

Understanding how soft matter behaves at the molecular level near the glass transition is of enormous scientific and practical importance.^1–3^ Kinetic freezing of a molecular system in a desired conformation for extended periods is particularly essential in nanotechnology, industry, and medicine.^1,4^ For this reason, the description of the out-of-equilibrium response of the glassy material has been a subject of intensive research for many decades.^2,5,6^ This challenge becomes even more pressing for glass-forming systems spatially confined at the nanoscale level as they fall out of equilibrium under conditions where their bulk counterparts do not. As a consequence, their properties may evolve substantially over a material’s service life. Predicting—rather than merely recording this evolution for an arbitrary thermal history is therefore of direct practical relevance. A natural starting point for such predictions is the theoretical framework developed over decades for the structural recovery of macroscopic glasses.

In the bulk state, a glassy material falls out of equilibrium when molecular mobility becomes too slow relative to experimental time scales. Out-of-equilibrium conditions occur below the glass transition temperature (*T*_g_) and result in time-dependent changes in material properties (such as density, enthalpy, refractive index, or Young’s modulus, etc.) as structural recovery takes place.^7–11^ In the 1970s, building on Tool’s notion of fictive temperature, Narayanaswamy formulated the concept of “material time”, according to which structural recovery is governed by an internal clock whose rate changes as the glass ages.^6,12–14^ In line with this concept, upon physical aging a glass evolves not according to the laboratory time, t, but according to its own internal time, termed as the material time *t̃*, and advances at a rate set by the instantaneous molecular mobility of the system. Formally, the material time links to laboratory time via the instantaneous relaxation rate τ­(*t*). When expressed in material time, the response of the glass becomes linear, that is, equal increments of *t̃* correspond to equal amounts of structural relaxation, regardless of how far the system is from equilibrium. As a matter of fact, Narayanaswamy “material time” concept underlies a family of models that differ in how the nonlinearity of aging—i.e., the dependence of the clock rate on the instantaneous state of the glass—is parametrized. In the widely used Tool–Narayanaswamy–Moynihan (TNM) formulation, the clock rate is expressed in terms of the fictive temperature. Alternative formulations invoke, for instance, the Adam–Gibbs relation or, more recently, density scaling.^11,15–17^ Within the single-parameter aging (SPA) approach, the nonlinearity takes its simplest form: the logarithm of the relaxation time (the clock rate) is assumed to depend linearly on the departure of a single monitored property from its equilibrium value.^18,19^ The TNM model has been successfully applied to describe structural recovery in numerous organic and inorganic glass-forming systems.^7,20–23^ However, it should be noted that it is physically valid only for temperature jumps within the structural recovery is governed by the α-relaxation, and in practice, corresponds to jumps terminating in the close vicinity of *T*_g_.^17–19,23^ For deeper quenches into the glassy state, more recent experimental observations indicate a multiple-step mechanism, in which contributions from more localized modes with lower activation energies must also be considered. This includes, for example, secondary relaxations of the Johari–Goldstein type or the recently identified slow Arrhenius process (SAP).^24–28^

Predicting the structural recovery of glassy materials within the framework of single-parameter aging (SPA) has a long tradition, dating back to the Tool–Narayanaswamy model and continuing through more recent contributions.^13,23,29–32^ It has also been demonstrated that this approach remains valid for describing equilibration phenomena observed in nanopore-confined glass-forming systems.^33,34^ In such confined geometries, the out-of-equilibrium behavior originates from the kinetic arrest of molecular mobility near the pore walls, which occurs at temperatures significantly higher than the glass transition temperature of the bulk material. The departure from equilibrium takes place far above the bulk *T*_g_, where the core fraction still retains high molecular mobility. Consequently, the structural recovery can be traced on laboratory time scales by monitoring changes in the α-relaxation time of the mobile core fraction. This is in contrast to the bulk, which falls out of equilibrium only below *T*_g_, where structural recovery becomes extremely slow. Therefore, equilibration kinetics for nanopore-confined glass-forming liquids can be observed on time scales that are 6 to 8 orders of magnitude faster than those of structural recovery in the bulk below *T*_g_.^35,36^

At a given temperature, soft matter systems confined within cylindrical nanopores typically exhibit faster dynamics than their bulk counterparts.^37–39^ This confinement regime is identified empirically as the temperature range below the vitrification temperature of the interfacial layer. The interfacial layer denotes here the fraction of molecules adsorbed at the pore walls due to strong attractive interactions. The dynamics of this layer is substantially slowed down relative to the pore interior, and it vitrifies at a temperature well above the bulk *T*_g_. When the interfacial layer becomes kinetically frozen, the α-relaxation time (τ_α_) of the confined liquid departs from the bulk behavior. Additionally, a second glass transition event can be detected in the calorimetric response of such nanopore-confined liquids.^40^ The thickness of the adsorbed interfacial layer in anodic aluminum oxide (AAO) nanopores remains a minor fraction of the pore diameter.^41^ We also note that the confinement effects investigated in the present study should be considered interfacial rather than geometric. The pore diameters are much larger than both the molecule size and the expected structural cooperativity length scale of the α-relaxation.^42^ Experimental evidence shows that extended annealing can eliminate confinement effects observed below the vitrification temperature of the interfacial layer and restore the α-relaxation time characteristic for a bulk material.^43–45^ The equilibration phenomena observed in nanopores do not represent classical physical aging of a glass below *T*_g_, but rather the equilibration of a confined liquid within the temperature window where its α-relaxation departs from the bulk-like VFT behavior. Its origin lies in the vitrification of the interfacial layer, which decouples the density of the pore interior from that of the bulk. Both phenomena—macroscopic glass aging and equilibration effects seen in nanopores—differ in the temperature range, origin, and accessible time scales but share characteristic features such as intrinsic isotherms, asymmetry of approach, and memory effects.^33,34^ The relaxation mode mediating the recovery in confinement is also the structural α-relaxation, which explains why the phenomenological material-time framework established for macroscopic glasses can be transferred to confined systems.

Within the single-parameter aging (SPA) framework, it is also possible to simplify the description of the equilibration phenomena observed in nanopores by replacing the experimentally measured time, t, with the so-called material time, *t̃*. SPA modeling implies that the generic normalized relaxation function *R* is a unique function of material time, introduced above. Therefore, all protocol-specific information, such as the annealing temperature, the magnitude, and the direction of the temperature jump, enters the description solely through the clock rate. As a result, once the reference relaxation function is established, the equilibration kinetics for any other temperature jump, pore size, or thermal history can be predicted by simply rescaling the clock. Interestingly, experimental data collected so far—primarily for polymer glass-formers confined in cylindrical nanopores—demonstrate that equilibration curves can be derived with remarkable accuracy from the reference relaxation function, even for temperature jumps as large as 50 K.^33^

This work extends previous studies of material-time scaling in nanoconfined glasses to 5-ring polyphenyl ether (5PPE), a simple, model van der Waals glass-forming liquid.^29,30^ 5PPE also belongs to the class of strongly correlated (single-parameter) liquids, i.e., a class of systems with strong correlations between virial and potential-energy fluctuations, whose dynamics on all time scales is governed by a single parameter, and it is invariant along isomorphs.^46^ This independently makes 5PPE a natural candidate for testing the single-parameter aging description in nanoscale confinement.

Here, our goal was to investigate out-of-equilibrium phenomena for 5PPE confined in anodic aluminum oxide (AAO) nanopores. We performed a series of time-dependent experiments at various annealing temperatures following temperature jumps of different magnitudes. Using dielectric spectroscopy (DS), we monitored the equilibration kinetics in the confined geometry and analyzed the collected data within the SPA framework. Our results show that the time required to eliminate the confinement effect depends on pore size, the specific thermal history, and the annealing temperature. The application of the SPA idea to nanopore-confined 5PPE yields the general observation that the model describes the approach to equilibrium relatively well, though in some cases, we encountered difficulties accurately predicting the initial stages of the recovery process.

## 2 Experimental Section

### 2.1 Materials

The tested sample is 5PPE (five-ring polyphenyl ether), a commercially used vacuum-diffusion pump fluid marketed under the brand name Santovac 5. The sample was purchased from Inland Vacuum Industries as a light yellow, essentially clear, viscous liquid and used as received. The value of *T*_g_ determined from differential scanning calorimetry is 250.5 K, while from the dielectric investigation we obtain 246.7 K (defined as the temperature at which the α-relaxation time is 100 s), which agrees well with the literature.^31,40^ The chemical structure of 5PPE is presented in Scheme 1.

**Scheme 1:**
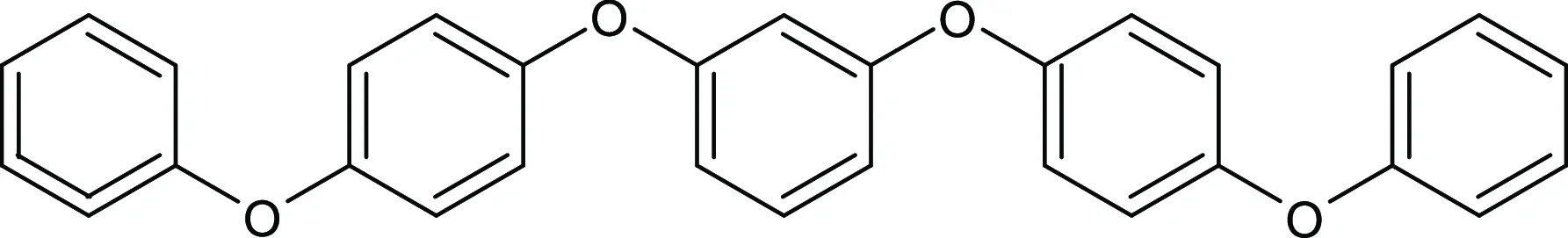
Chemical Structure of 5PPE

We used commercially available anodized alumina (AAO) membranes (InRedox, Inc.) as confining templates. They are composed of uniform arrays of unidirectional, uncross-linked nanochannels, aligned perpendicular to the material’s surface and penetrating its entire thickness. The diameter of each membrane is 13 mm, while its thickness is 100 μm. In the present study, we have used alumina membranes with the following pore sizes: 40, 80, 100, 120, and 150 nm. The membranes’ porosity ranges from 12% to 15% (depending on pore size). The pore size distribution varies within ±5 nm. Before infiltration, AAO templates were dried at 453 K for 24 h in a vacuum oven to remove volatile contaminants from the nanochannels. Dielectric spectra were collected for each empty membrane within the same temperature range as for the bulk material. Then, they were placed in small containers filled with 5PPE. The infiltration procedure was performed at 333 K for at least 2 weeks under vacuum to allow liquid to flow into the nanopores by capillary forces. Membranes were weighed before and during the infiltration process. Following infiltration, any excess liquid was removed by carefully wiping the AAO surface with low-lint, pure-cellulose laboratory wipes designed for cleaning optical components and no fiber residues were confirmed under the optical microscope. The infiltration was assumed to be complete when the mass of the filled membrane no longer changed with infiltration time. Based on membrane porosity, liquid density, and the membrane’s mass before and after infiltration, it is possible to estimate the percentage of pore filling.^47,48^ In our case, the filling fractions were 75 ± 15% (40 nm), 85 ± 15% (80 nm), 87 ± 15% (100 nm), 83 ± 15% (120 nm), and 90 ± 15% (150 nm).

### 2.2 Methods

Dielectric relaxation measurements were performed using an Alpha-A impedance analyzer equipped with a Novocool temperature control system (Novocontrol, Germany). For bulk sample measurements, we have used 10 mm-diameter stainless steel electrodes, separated by a 50 μm Kapton ring to maintain a fixed distance between the plates. To investigate nanopore-confined samples, the same parallel-plate geometry with 10 mm diameter electrodes was employed. Dielectric spectra for the bulk liquid were collected over the temperature range from 293 to 233 K during cooling in 2 K steps. For liquid-infiltrated AAO templates, we also collected dielectric loss spectra in the temperature range from 233 to 293 K and used these results to determine the temperature dependence of α-relaxation time. The thermal protocol was, however, slightly different than for a bulk liquid, and involved cooling to 233 K, the lowest examined temperature, followed by heating in 2 K steps up to 293 K. In both cases, the temperature was stable to within 0.1 K. The complex dielectric function, ε* = ε′ – *i*ε″, where ε ′ and ε′′ are the real and the imaginary parts, was recorded in the frequency range from 0.05 Hz to 1 MHz.

Time-dependent experiments were carried out using the same experimental setup, following a series of thermal protocols that involved either downward or upward temperature jumps of varying magnitudes (4, 6, and 8 K). Before performing any temperature jump from the intermediate temperature to the target annealing temperature, we always ensured that the nanopore-confined system was in equilibrium. Equilibrium state was verified by monitoring changes in the position of the α-relaxation peak over time. In some cases, reaching equilibrium at the intermediate temperature, i.e., the initial temperature from which the jump was made, required waiting for approximately 2–3 days. Only after that, the actual temperature jump was performed. Dielectric spectra were collected continuously over the frequency range from 0.1 Hz to 1 MHz. Importantly, the time required to reach the desired annealing temperature did not exceed 2–3 min; the stabilization period was not included in the analyzed data. Control jumps performed on bulk 5PPE under identical conditions show that τ_α_ attains its new equilibrium value in the first spectrum recorded after the jump, confirming that thermal equilibration of the sample should not distort the shape of the recovery curves. A single spectrum was collected within less than 120 s, which is fast enough to avoid significant structural changes and capture the maximum of the α-loss peak. For the analysis of equilibration kinetics, time zero was defined as the time when the sample temperature had reached the target annealing temperature to within ±0.2 K. The equilibration process was considered complete when the α-relaxation time no longer changed with time. Scheme 2 illustrates the thermal protocol of the equilibration experiments carried out in this study. The measured dielectric spectra were corrected by modeling the system as two capacitors connected in parallel—one representing the signal from the confined liquid and the other corresponding to the porous matrix.^49–51^ The assumptions and sensitivity of this procedure have been analyzed in detail ih the cited works. Importantly, the correction affects only the amplitude of the dielectric response, whereas the position of the α-relaxation peak-the sole quantity on which the present analysis is based—is insensitive to it.

**Scheme 2:**
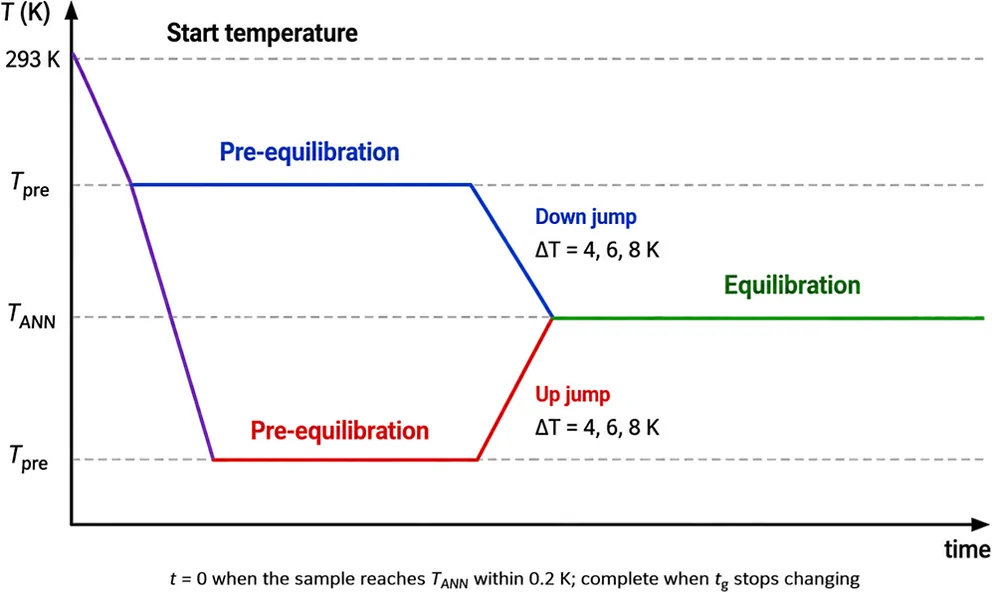
Schematic View of the Thermal Protocol of the Equilibration Experiments Carried out for 5PPE in Nanopores^a^

a The starting temperature is always 293 K. From this temperature, we cool the sample to the pre-annealing temperature *T*_pre_ and equilibrate until τα remains constant (up to 2–3 days). A temperature jumps of |Δ*T*| = 4, 6, or 8 K—down (blue) or up (red)–are then performed to the annealing temperature *T*_ANN_, and dielectric spectra are recorded every 120 s (green) until τα ceases to change. Time zero is defined as the moment the sample temperature reaches *T*_ANN_ within 0.2 K.

## 3 Results and Discussion

Multiple studies demonstrate that the dynamics of glass-forming systems in nanopores can deviate significantly from bulk behavior.^52,53^ In alumina and silica nanopores, the most common observation is that molecular mobility related to the glass transition is faster than in the bulk. More specifically, decreasing pore size markedly enhances cooperative dynamics.^54^ However, the α-relaxation dynamics in confined geometry also depend strongly on the thermal protocol and evolve with time.^44,45^ Therefore, prolonged annealing of nanoconfined glass-formers can restore their bulk-like dynamics.^43,55^ Both features are commonly discussed in the context of density frustration within the pore interior or adsorption–desorption (molecular exchange) processes occurring near the pore surface that allow this deviation to relax.

Our focus here is the analysis of equilibration kinetics in nanopores and the description of structural recovery using the existing approaches previously employed to model physical aging of bulk glasses. We followed equilibration kinetics by dielectric spectroscopy and analyzed the time evolution of the α-relaxation time. The structural (α-) relaxation originates from cooperative molecular motions and is the dominant relaxation mode observed in the temperature range where the nanopore-confined sample reveals out-of-equilibrium behavior. Figure 1 (top and bottom) demonstrates changes of the α-relaxation for 5PPE confined in 80 nm pores at *T*_ANN_ = 257 K after up and down jumps of the same magnitude, Δ*T* = ±4 K, respectively. As shown, the position of the α-relaxation peak changes over time, indicating that out-of-equilibrium phenomena in nanopores occur at temperatures well above those for the bulk glass. In the down-jump experiments, we observed a shift in the α-relaxation toward lower frequencies, indicating a slowing of molecular mobility over time. In contrast, for up jumps, the α-loss peak shifts to higher frequencies, indicating that molecular mobility increases as the system approaches equilibrium. The initial equilibrium states were achieved by preannealing of the confined liquid at either *T*_pre_ = 253 K or *T*_pre_ = 261 K. In the supercooled liquid state, the time scale of structural relaxation changes from milliseconds to seconds. While for a bulk liquid this is still fast enough to reach equilibrium after a temperature jump, this is not the case for the nanopore-confined sample. Therefore, it falls out of equilibrium at much faster time-scales.

**Figure 1:**
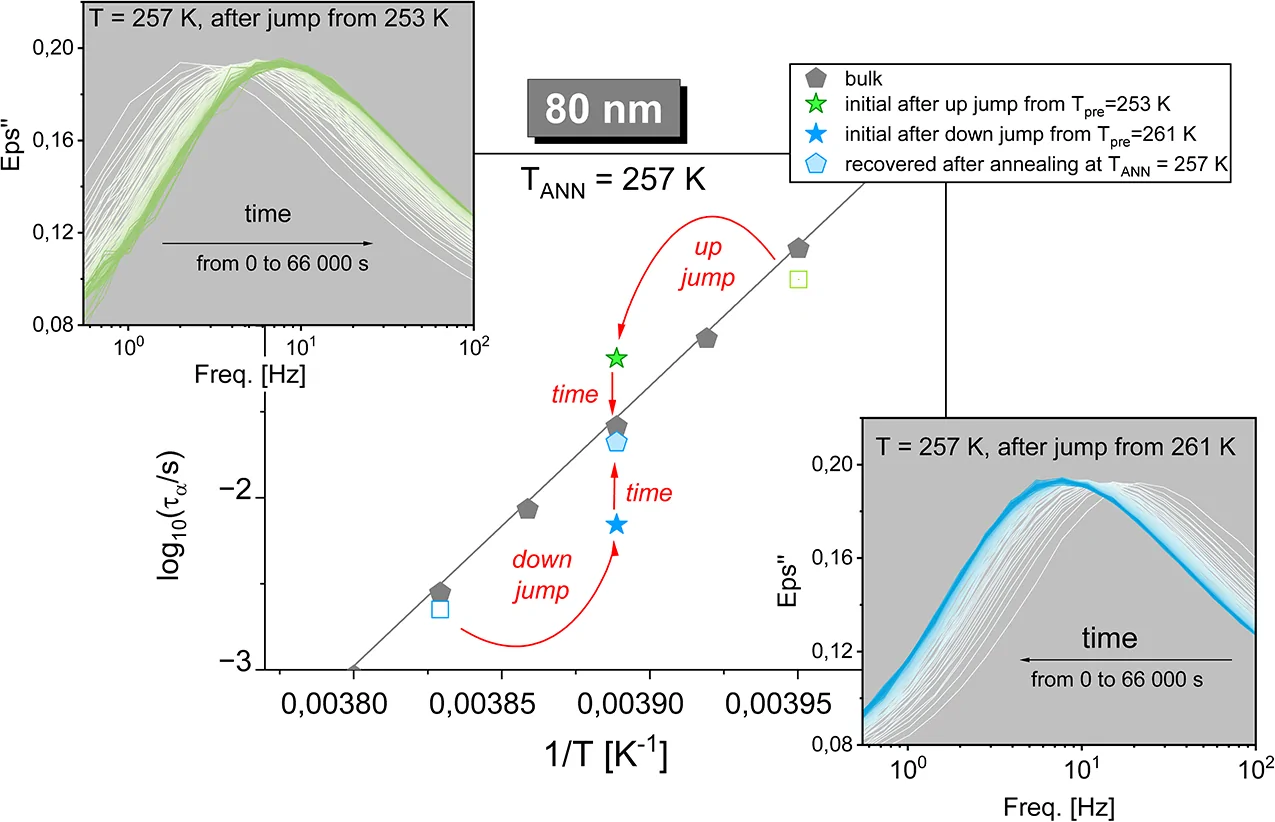
Temperature dependence of the segmental relaxation time for 5PPE in bulk and confined in 80 nm AAO nanopores. The α-relaxation times for the confined samples were recorded after temperature jumps from *T*_pre_ = 253 K and *T*_pre_ = 261 K to *T*_ANN_ = 257 K (Δ*T* = ± 4 K). The solid line represents the fit of the bulk data to the Vogel–Fulcher–Tammann (VFT) equation which provides the equilibrium bulk τ_α_ within the temperature range that covers all annealing temperatures investigated in this work. VFT interpolated values define the equilibrium levels toward which the recovery in confinement converges. The top and bottom graphs show the shift in the α-relaxation for 5PPE confined in 80 nm pores upon annealing at 257 K.

From the position of the maximum of the loss peak (*f*_max_), we determined the α-relaxation time $\left(\tau_{\alpha }=\frac{1}{2\pi f_{\max}}\right)$ and plotted it together with the bulk data in Figure 1 (main graph). As shown, nanopore-confined material subjected to a temperature jump can recover its bulk relaxation time when waiting long enough. Reaching equilibrium here means that the averaged α-relaxation time of the confined material becomes comparable to that of the bulk material at the same temperature. For 5PPE confined in 80 nm AAO, the annealing time required to go from nonequilibrium to equilibrium state takes ∼10 h. It is worth reminding that, for a bulk glass, it is not possible to directly measure the relaxation time in the equilibrium state at temperatures far below the glass transition temperature because the aging time would be impractically long.^7,20^

Figure 2a shows the initial values of the α-relaxation times for 5PPE confined in pores of different sizes, collected at the same annealing temperature, *T*_ANN_ = 257 K, after up and down temperature jumps of the same magnitude (Δ*T* = ±4 K). For the down-jump experiment, the cooperative mobility recorded at the initial stages of the annealing process is enhanced compared to the bulk counterpart as the pore size decreases. This observation remains consistent with the conventional understanding that the relaxation dynamics accelerate due to a systematic decrease in the packing density of the molecules as the pore size is reduced.^38^ The up-jump experiments are fully consistent with this picture: in both cases, the dynamics at the beginning of annealing is faster in smaller pores. However, when expressed as the distance from the equilibrium τ_α_, the initial departure appears below the bulk value for down jumps and above it for up jumps. Moreover, for up jumps it shows the opposite pore-size trend than for down jumps. So far, we do not have a firm explanation for this finding, but we suppose this trend might be related to the interfacial effects and different rates of the molecular exchange between the core and the interfacial layer—a quantity that scales with the surface-to-volume ratio of the pore—when following various thermal protocols.^41^ This interpretation could be tested in the future, e.g., by modifying the pore-surface chemistry at fixed pore geometry, which should alter the exchange kinetics without changing the confinement length scale. The results presented in Figure 2a also show that prolonged annealing in nanopores erases faster dynamics and recovers τ_α_ characteristic for the bulk liquid.

**Figure 2:**
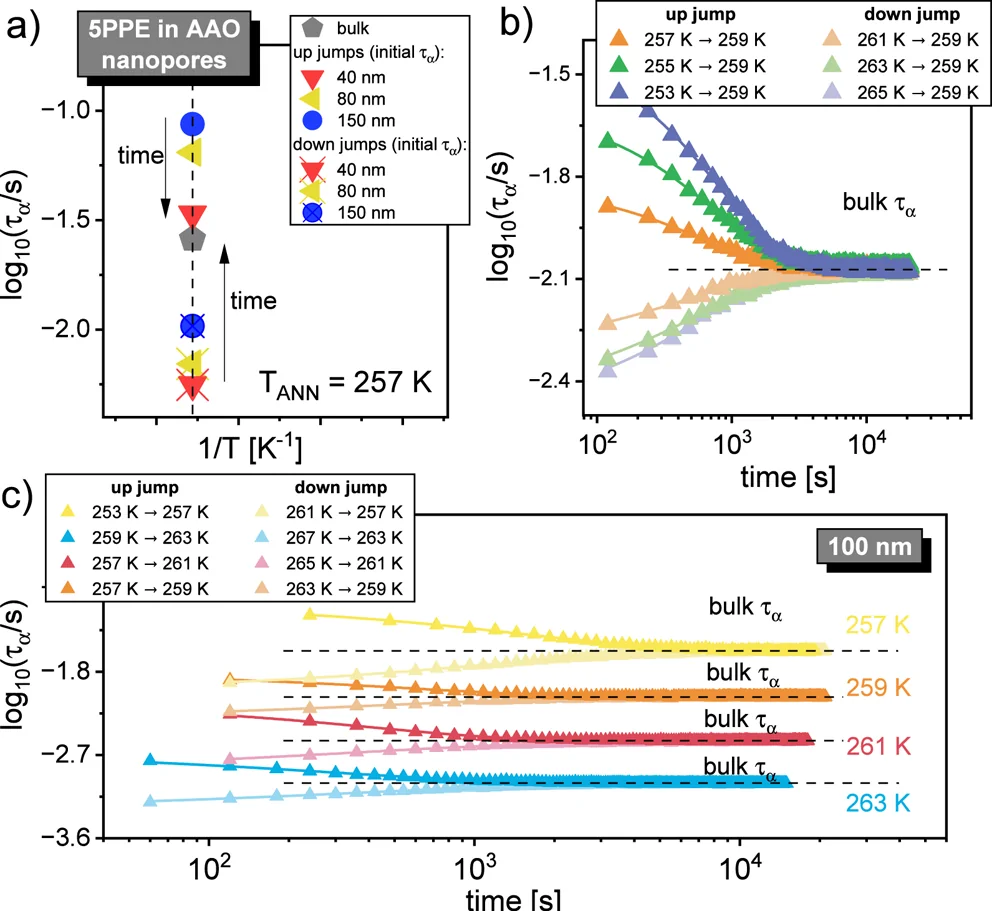
(a) Initial values of the α-relaxation time for 5PPE in bulk and confined in nanopores with different pore sizes collected at *T*_ANN_ = 257 K just after up and down jumps from *T*_pre_ = 253 K and *T*_pre_ = 261 K, respectively. (b,c) Evolution of α-relaxation as a function of time for 5PPE confined in 100 nm alumina nanopores for various up and down temperature jumps, as listed in the legend. Solid lines show fits of the experimental data to a modified version of the stretched exponential function.

Within the conventional description of physical aging, one of the most fundamental signatures of the structural recovery process is its nonlinearity. The nonlinearity of physical aging arises from the fact that the relaxation time of the molecular mechanisms governing the equilibration process changes with the thermodynamic state of the glass.^7,27^ In a typical aging experiment, this is demonstrated by the so-called “asymmetry approach”. The thermal protocol for the asymmetry-of-approach experiment involves following the equilibration kinetics at the same temperature *T* for two alternative up/down temperature jumps, both starting from the equilibrium state and of the same magnitude, *T* ± Δ*T*. Figure 2b,c show results from the asymmetry-of-approach experiment conducted for 5PPE confined in AAO nanopores. Similar to what was reported for bulk glasses, we also found that the two equilibration curves, for up and down temperature jumps, are not symmetric and do not exhibit the same magnitude of departure from the equilibrium state, especially at short times. Moreover, for down jumps, the response is somewhat faster at the beginning of the equilibration process because the system starts from a higher-mobility state. In contrast, for up jumps, the initially confined material has lower mobility compared to the equilibrium state, but it self-accelerates over time. The set of experimental data presented in Figure 2b shows that for a larger temperature jump, the initial departure from bulk behavior increases. In contrast, the time required to reach equilibrium is essentially controlled by the annealing temperature. The jump magnitude produces a modest, systematic increase in the characteristic recovery time constant τ_ANN_ (for both up- and down-jumps), which we attribute to the nonlinearity of physical aging. Likewise, decreasing the annealing temperature slows down the equilibration process in nanopores (see Figure 2c).

Three distinct time scales appear in the analysis that follows: (i) the structural α-relaxation time which is the experimentally monitored quantity, τ_α_, (ii) the annealing (recovery) time constant, τ_ANN_, which quantifies how long it is needed to reach equilibrium at a given temperature and protocol, and (iii) the material time, *t̃*, which is not a measured quantity but an internal variable that reweights the laboratory time by the clock rate. To analyze the time scale of equilibration phenomena observed in pores of different sizes and under various thermal protocols, we described all collected τ_α_(*t*) data using a modified version of a stretched exponential function

1 $$\tau_{\alpha }=A\exp{\left(-\frac{t}{\tau_{ANN}}\right)}^{\beta }+B$$

where *A* is the prefactor, τ_ANN_ decay time constant, β stretching exponent, while *B* the value of the measured quantity at infinite times. The best fits of eq 1 are displayed in Figure 2b,c as solid lines. Table 1 collects characteristic annealing time constants and stretching exponents. The pronounced asymmetry between down and up jump experiments also manifests in a lower stretching exponent for the former one (β ≈ 0.56–0.67 versus 0.69–0.83) suggesting it depends on the initial structural state. Within the material-time concept, such an asymmetry arises naturally from the nonlinearity of the equilibration process as the internal clock evolves in opposite directions for up and down jumps. The stretching exponent β shows no systematic dependence on pore size, but the characteristic time constant τ_ANN_ decreases monotonically with increasing pore diameter for both jump directions. Moreover, the pore-size dispersity does not account for the stretching: β shows no systematic decrease toward smaller pores, where the relative dispersity is largest.

**Table 1:** Characteristic Annealing Time and Stretching Exponents for 5PPE Confined in AAO Nanopores as Determined by Fitting Experimental Data to eq 1

characteristic annealing time (log_10_ τ_ANN_/[s])					β ± error			
up jumps	40 nm	80 nm	100 nm	150 nm	40 nm	80 nm	100 nm	150 nm
257 K → 261 K			2.61 ± 0.01				0.81 ± 0.02	
255 K → 259 K			2.85 ± 0.01				0.83 ± 0.01	
253 K → 257 K	3.88 ± 0.01	3.33 ± 0.01	3.18 ± 0.01	3.02 ± 0.01	0.76 ± 0.01	0.69 ± 0.02	0.75 ± 0.02	0.75 ± 0.01

characteristic annealing time (log_10_ τ_ANN_/[s])					β ± error			
down jumps	40 nm	80 nm	100 nm	150 nm	40 nm	80 nm	100 nm	150 nm
265 K → 261 K			2.55 ± 0.01				0.56 ± 0.01	
263 K → 259 K			2.70 ± 0.01				0.61 ± 0.01	
261 K → 257 K	3.67 ± 0.02	3.28 ± 0.01	3.01 ± 0.01	2.90 ± 0.01	0.56 ± 0.02	0.65 ± 0.01	0.67 ± 0.01	0.61 ± 0.01

To analyze equilibration data across different thermal paths, we define the normalized relaxation function varying between 0 and 1. In general form, it is given as

2 $$R\left(t\right)=\frac{X\left(t\right)-X_{eq}\left(T\right)}{\Delta X\left(0\right)}$$

where Δ*X*(0) is the total change of the measured property from initial to equilibrium states, *X*_eq_(*t*) the equilibrium value of the followed property, and *X*(*t*) a time-dependent property used to track equilibration progress. In our case, the property monitored during the equilibration process is log_10_τ_α_(t). Figure 3a shows *R*(*t*) data for 5PPE confined in pores of different sizes collected at the same annealing temperature, after up and down jumps of the same magnitude. As observed, the *R*(*t*) dependence has a sigmoidal shape and shifts to longer aging times with decreasing pore size. It also becomes more stretched as pore sizes decrease. Figure 3b collects *R*(*t*) in 100 nm pores after 4 K temperature jumps to different temperatures. As the annealing temperature decreases, the equilibration time increases, and the *R*(*t*) curves shift to longer times.

**Figure 3:**
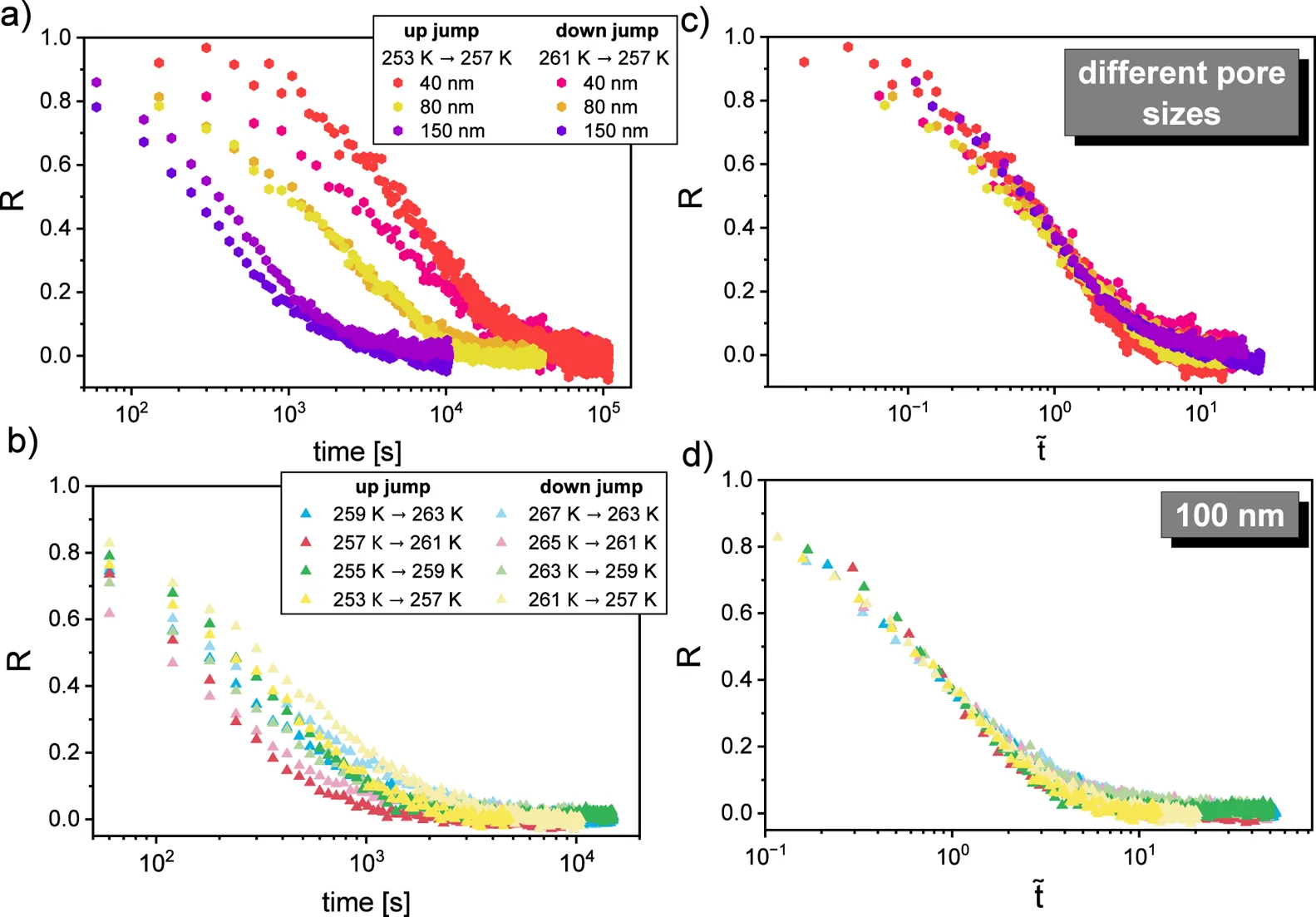
Normalized relaxation function for various down jumps plotted versus laboratory time (a,b) and material time, *t̃* (c,d) for 5PPE confined in AAO nanopores. Graph (a) shows *R*(*t*) at *T*_ANN_ = 257 K for 5PPE confined in pores of different sizes, while (b) *R*(*t*) dependences in pores of the same size (100 nm) but annealed at different temperatures. The corresponding data presented in terms of material time are shown in (c,d), respectively.

In line with the classical description of structural recovery, we assume in the following that the α-relaxation dominates the equilibration process. This can be rationalized by the fact that we performed small temperature jumps within the temperature range where the α-relaxation governs the confined liquid’s dynamics. Under this assumption, the effect of nonlinearity can be quantified employing the concept of material time, *t̃*, related to the clock rate γ­(t), that is, the inverse of the relaxation time

3 $$d\mathrm{t̃}=\gamma \left(t\right)dt=\frac{1}{\tau \left(t\right)}dt$$

In this way, the nonlinear response can be reduced to the linear response at equilibrium by simply replacing the time, t, with the material time, *t̃*. The material time formalism predicts that for all temperature jumps the normalized relaxation function is a unique function of the material time. In this way, experimental data superimpose onto a single curve when plotted as a function of material time. The experimental evidence that the material-time concept also applies to the description of the equilibration phenomena observed in nanopore confinement is demonstrated in Figure 3c,d. As can be seen, all the *R*(*t*) dependences collapse within the experimental uncertainty (RMS deviation from a common stretched-exponential master curve of ∼0.023 in units of *R*, comparable to the plateau scatter of the individual curves). In the case of equilibration phenomena that we study in pores, the time-dependent characteristic time was approached by aging (or annealing) time decay constant determined from the fit of experimental data to eq 1.^56,57^ Further evidence of the validity of the material time concept applied to describe and predict equilibration phenomena in nanopores will be given in terms of the single parameter aging concept (SPA).

We stress a conceptual simplification specific to the present experiments. In classical structural-recovery studies, the aging is followed through a secondary property (volume, enthalpy, or the low-frequency permittivity). In such a case, the single-parameter (SPA) assumption is required to relate the evolution of the measured property to the underlying relaxation time that governs the clock rate. In nanopore confinement, by contrast, the quantity that we follow upon equilibration is the α-relaxation time itself, *X*(*t*) = log_10_ τ_α_(*t*). So, the clock rate is accessed directly from the experimental data—here through the time-dependent annealing time constant extracted from *R*(*t*). Consequently, the single-parameter link between an arbitrary monitored property and the relaxation time, which is an essential ingredient of SPA for conventional observables, is not required in our analysis. The collapse of all *R*(*t*) curves onto a common master curve (Figure 3c,d) confirms, however, that a single material time governs equilibration across different jump magnitudes, pore sizes, and directions and that at least within the temperature window where the α-relaxation dominates the recovery.

SPA is a model-dependent analysis of aging data based on the reformulation of the Tool-Narayanaswamy idea of the material time.^17–19,23^ It follows the assumption that any aging system has an internal clock that ticks at a rate that depends solely on a single nonequilibrium parameter that controls all other properties of the aging materials, including the property being monitored during aging. If obeyed, the SPA phenomenology has been shown to describe the equilibration of aged glasses for temperature jumps as large as 20 K,^17^ and, predict a whole family of relaxation curves from a single reference curve.^17,19,23^ As mentioned earlier, it has also been successfully tested for polymer glass-formers confined in alumina nanopores, while this work provides further evidence.

If the TNM formalism applies, *R* can be treated as an independent variable, while time is the dependent variable. In a very condensed form, a general recipe which can be used to predict a set of experimental data measured after an arbitrary temperature jump *B* from another set of experimental data points corresponding to a different temperature jump *A* is given as

4 $$t_{B}\left(R\right)=\frac{\gamma_{eq,A}}{\gamma_{eq,B}}\int_{0}^{t_{A}}\exp\left(c\frac{X_{A}\left(0\right)-X_{B}\left(0\right)}{X_{eq}}R_{A}\left(t_{A}\right)\right){dt}_{A}\left(R\right)$$

where γ_eq,A_ and γ_eq,B_ are equilibrium values of the clock rates, *c* is a dimensionless constant connecting the monitored quantity to the material clock rate, *X*_A_(0) and *X*_B_(0) denote the total change of the measured quantity for jumps *A* and *B*, while *X*_eq_ is the equilibrium value of the measured property. Thus, within the SPA approach for any temperature jump, it is possible to convert the time axis from one nonlinear jump to another.^18,19^ We applied eq 4 to predict annealing curves for the studied molecular liquid confined in nanopores of different pore sizes and subjected to various thermal protocols. The results of this analysis are shown in Figure 4. Dashed lines show the model’s prediction, while symbols represent measured data. As shown, the model predicts equilibration curves very well, not only for corresponding up-and-down jumps collected in pores of the same size, but also for pores of different sizes. To reconstruct the shape of the entire curve, the experimental data were fitted with a stretched exponential, which helped interpolate between data points. Then, the fitted curve was used to generate the other temperature jump using eq 4. For 5PPE confined in alumina nanopores, *c* = 1 because we can access the material clock rate directly from the experiment. We verified this value also from the data using the self-consistency condition applied to pairs of up- and down-jumps ending at the same temperature.^18,19^ The model shows deviations from the experimental values, especially at the initial stages of equilibration, as the predicted curves are consistently below the data points. Part of this discrepancy may be due to the scattering of the experimental data and the difficulty in following equilibration kinetics at shorter times. The finite time resolution of the measurement (spectra collected every 120 s) restricts the reliability of the very first points of the recovery curves and may contribute to the deviations observed at the initial stages of the recovery process. A systematic deviation seen at short time may, however, also carry physical information. Therefore, a plausible contribution coming from additional mechanisms such as interfacial rearrangements near the vitrified adsorbed layer or more localized relaxation modes cannot be excluded. The present data do not allow us to discriminate between these possibilities. We note that the quality of the prediction within eq 4 is not inferior to the quality of predictions reported for macroscopic glasses.^18,19^ A dedicated study of the short-time regime, with improved time resolution, would be required to settle this point.

**Figure 4:**
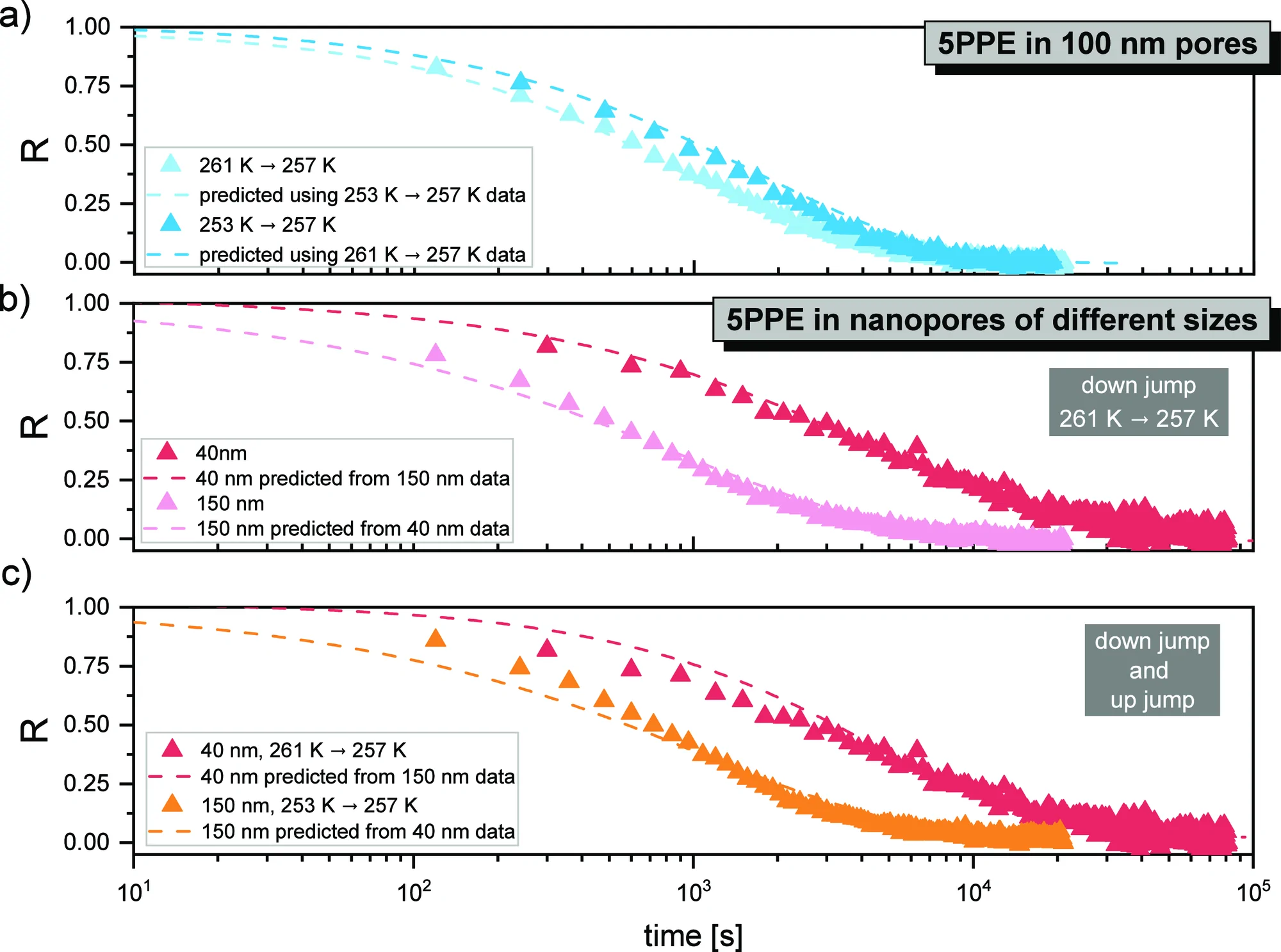
Experimental (points) and predicted (lines) normalized relaxation function vs annealing time for 5PPE confined in AAO nanopores. All annealing experiments were performed at the same temperature, *T*_ANN_ = 257 K (with a depth of each step of Δ*T* = ± 4 K). Panel (a) shows up- and down-temperature jumps obtained in 100 nm pores, while (b,c) show predictions for various jumps in nanopores of different sizes (40 and 150 nm).

In recent years, it has been suggested that the SPA description of physical aging in polymer glasses might be inadequate, especially when modeling equilibration kinetics for large temperature jumps, because of contributions from other molecular mechanisms beyond the α-relaxation.^24–27^ In such a case, analysis of the effective activation energy as a function of conversion has led to the conclusion that more localized, low-energy-barrier molecular processes mediate the initial stages of aging. At the same time, the α-relaxation dominates the final approach to equilibrium. A very useful tool to demonstrate this is the isoconversional approach, which allows evaluating the kinetics of a relaxation or transformation process by applying individual Arrhenius equations at different degrees of conversion.^25^ Here, we have applied the isoconversional method to describe equilibration phenomena seen in nanopores. This type of analysis requires performing a series of annealing experiments at different temperatures. Then, the data must be converted into curves of conversion vs time. For a series of temperature jumps, the isoconversion state would be ascribed to the same extent of conversion (*R*) and analyzed using a distinct Arrhenius equation. The isoconversional method assumes a single-step kinetic pathway, i.e., that at a given degree of conversion the system passes through an equivalent structural state irrespective of the annealing temperature. This condition is satisfied by our protocol: each experiment in the series starts from the equilibrium state of the confined liquid—a state uniquely defined by temperature, with no dependence on thermal history—and involves a down jump of the same magnitude (Δ*T* = 4 K), so that the initial departure from equilibrium is closely comparable across the set and only the absolute temperature of the recovery window varies. The relatively low activation barrier for equilibration phenomena observed in confined space should first be attributed to the role of the α-relaxation in nonequilibrium conditions. Therefore, in Figure 5d, we demonstrate the temperature evolution of the α-relaxation time for 5PPE confined in AAO nanopores of different sizes. In this case, the thermal protocol involved cooling the confined sample with >10 K/min to the glassy state and recording dielectric spectra every 2 K on heating. As can be observed, τ_α_(*T*) dependence follows bulk behavior only at higher temperatures. At lower temperatures, it deviates from the bulk. The characteristic kink in τ_α_(*T*) is commonly ascribed to vitrification of the molecules adsorbed to the pore walls and depends on the pore size.^40,41^ Therefore, out-of-equilibration phenomena in nanopore confinement span only within a temperature range where the dynamics of the interfacial layer become seemingly frozen in the time scale of the experiment. As mentioned earlier, this temperature can be defined either from calorimetric data (higher *T*_g_ value) or dielectric data—as the crossing point between the bulk-like VFT and Arrhenius-like dependence.^40^ The apparent activation energy of the confined α-relaxation time, determined in the temperature range below *T*_g_ of the interfacial layer, varies between 112 and 123 kJ/mol depending on the pore size and agrees, within the experimental uncertainty, with the activation barrier obtained from the isoconversional analysis of the equilibration kinetics (∼100 kJ/mol). A nearly constant activation energy across different degrees of conversion might indicate that a single mechanism mediates the entire equilibration process.^25,26^ We note, however, that the isoconversional barrier carries a sizable uncertainty that also encompasses the value estimated for SAP from the model proposed by White, Napolitano, and Lipson (*E*_SAP_ ≈ 67 ± 10 kJ/mol)^58^ with isobaric thermal-expansion coefficient α = 6.5 × 10^–4^ K^–1^.^59^ Therefore, on the basis of the present data, we cannot exclude a SAP contribution to the equilibration kinetics in confinement, especially at the latest stages.

**Figure 5:**
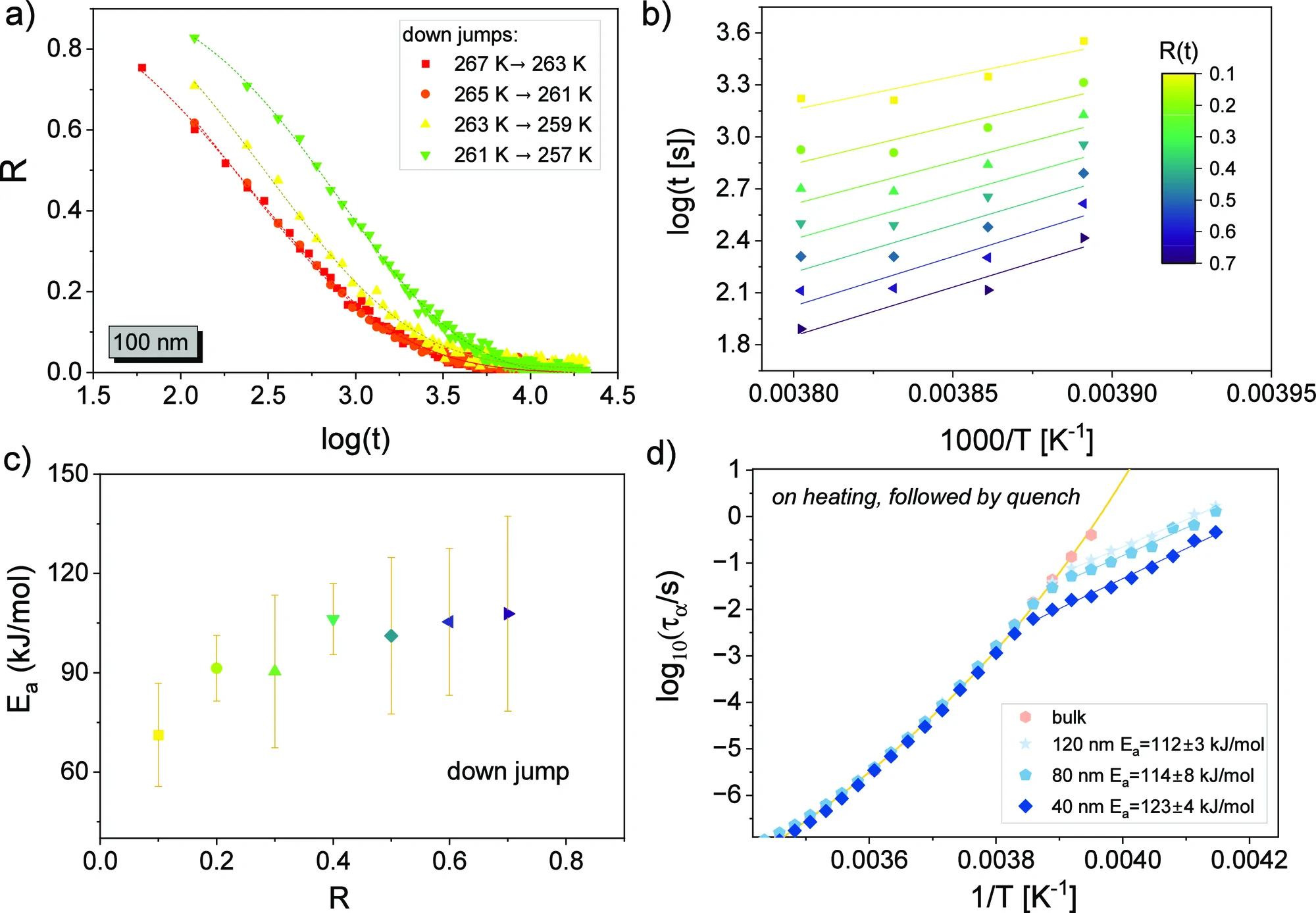
(a) Experimental data of the normalized relaxation function *R*(*t*) collected for 5PPE confined in 100 nm pores at the indicated temperatures after 4 K down jumps. Dashed lines represent the fit of the data to a modified version of the stretched exponential function. (b) Logarithm of the experimentally measured time necessary to reach a certain degree of conversion plotted as a function of the inverse temperature. (c) Activation barrier determined from isoconversional analysis for certain degrees of conversion. (d) Temperature evolution of α-relaxation time measured for 5PPE confined in pores of different sizes. Experimental data collected in the temperature range where deviation from the bulk dependence was observed were fit to the Arrhenius equation. Bulk data are shown as a reference.

## 4 Conclusion

In summary, we have investigated equilibration phenomena of nanopore-confined glass-forming liquid 5PPE within the material time concept. The material-time framework has been successfully applied so far only to describe the physical aging of numerous bulk glasses. This study provides new experimental evidence that the molecular origin of out-of-equilibrium phenomena observed in nanopores is very different and occurs at much different time scales than the physical aging of bulk glasses. Nevertheless, both processes share very similar features, including the ability to model temperature-jump relaxation curves from an arbitrary measured temperature jump. By applying isoconversional analysis to a set of equilibration data measured in confinement, we show that, within experimental uncertainty, the different stages of the equilibration process can be described by a single activation energy. For 5PPE confined in 100 nm AAO pores, the activation energy was ∼100 kJ/mol for the down jump. This value seems to correlate quite well with the activation energy for the α-relaxation measured within the same out-of-equilibrium conditions. However, it does not exclude the fact that the equilibration mechanism in confinement could also be affected by other processes of a more local origin, such as SAP.

Our results reinforce the view that out-of-equilibrium phenomena in nanopore confinement can be mapped out using the same concept as physical aging of bulk glasses. Taken together, the application of the material time concept and the SPA approach appears to be an effective tool for modeling and exploring equilibration phenomena in nanopores, which is warranted to build a comprehensive picture of the behavior of soft matter systems at the nanoscale.
